# Supplementary material for: Optical quality of Fresnel-structured hyperopic implantable collamer lenses: A ray-tracing analysis
Source: PLoS One. 2026 Apr 7;21(4):e0345116. doi: 10.1371/journal.pone.0345116 (PMC13056176; doi:10.1371/journal.pone.0345116)
Supplement: S1 File — (PDF) [file pone.0345116.s001.pdf]

Supporting Information

S1 File. Analysis of tuned Fresnel step-height conditions.

S1. Background and purpose

In Fresnel-type stepped optical structures, phase discontinuities at step edges may introduce diffraction-related effects. In particular, if the optical path difference between adjacent zones is not controlled, partial destructive interference may occur at the design wavelength. To address this concern, an additional analysis was performed in which the Fresnel step heights were adjusted to satisfy a tuned condition, where the optical path difference between adjacent zones corresponds to an integer multiple of the design wavelength.

This supporting analysis was conducted to examine whether enforcing such tuned step-height conditions influences the optical performance of the Fresnel-inspired hyperopic ICL, and to verify the robustness of the main conclusions presented in the manuscript.

S2. Tuned Fresnel step-height condition

The tuned Fresnel condition was defined such that the optical path difference (OPD) introduced by each step height satisfied:

$$\mathrm{OPD} = (n_{\mathrm{ICL}} - n_{\mathrm{aq}}) \cdot h = m\,\lambda_0$$

where n_{ICL} is the refractive index of the ICL material, n_{aq} is the refractive index of the surrounding aqueous humor, h is the step height, λ_0 is the design wavelength, and m is an integer. In this analysis, the design wavelength was set to λ_0 = 555 nm.

Based on these conditions, the step height was adjusted to satisfy the integer-wavelength optical phase difference (OPD) value at the design wavelength. Additional Fresnel ICL models were generated while maintaining all other design parameters identical to the original stepped Fresnel ICL (Tables 1 and 2).

Table1. Optical path difference (OPD) and corresponding phase lag at 555 nm for the step heights used in this study.

Step height h (μm) in this paper	OPD (μm)	Phase lag (λ)
25	2.85	5.14λ
50	5.70	10.27λ
100	11.40	20.54λ

Table 2. Relationship between integer-wavelength tuned step heights and the step heights used in this study at 555 nm.

integer m	Phase lag (λ)	tuned step height h_{tune} (μm)	step height h (μm) in this paper
1	1λ	4.87	-
5	5λ	24.3	25
10	10λ	48.7	50
20	20λ	97.4	100

To maintain design consistency, the second-order even-asphere coefficient was fixed at 0.025 for all tuned and non-tuned step-height conditions, and the fourth-order term was set to zero. By keeping the aspheric coefficient constant, the effects of step height and phase adjustment on optical performance can be compared and separated without introducing additional optimization degrees of freedom.

S3. Optical simulation conditions

Optical performance under the tuned step-height conditions was evaluated using the same simulation framework and analysis procedures as described in the main manuscript. The image-plane MTF was calculated using an FFT-based wave-optical method under both monochromatic illumination at 555 nm and polychromatic (white-light) conditions. For the polychromatic analysis, the same wavelength set and photopic weighting scheme as in the main manuscript were applied. Material dispersion was modeled using a representative Abbe number of 50. The pupil diameter, sampling resolution, and evaluation metrics were kept identical to those used in the primary analysis to ensure direct comparability.

S4. Results under tuned conditions

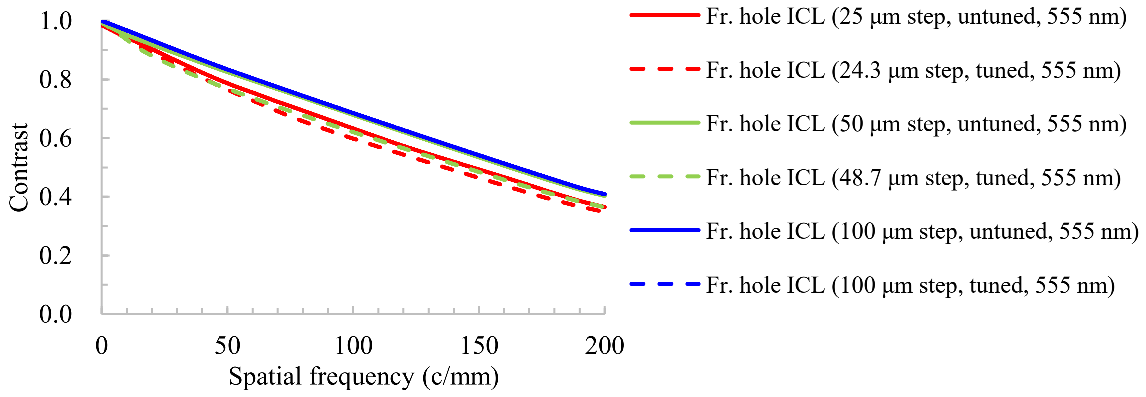

S1 Fig 1. FFT-based MTF comparison of Fresnel-inspired hole ICLs with different step heights under tuned and non-tuned conditions at a 3.0 mm artificial pupil (555 nm).

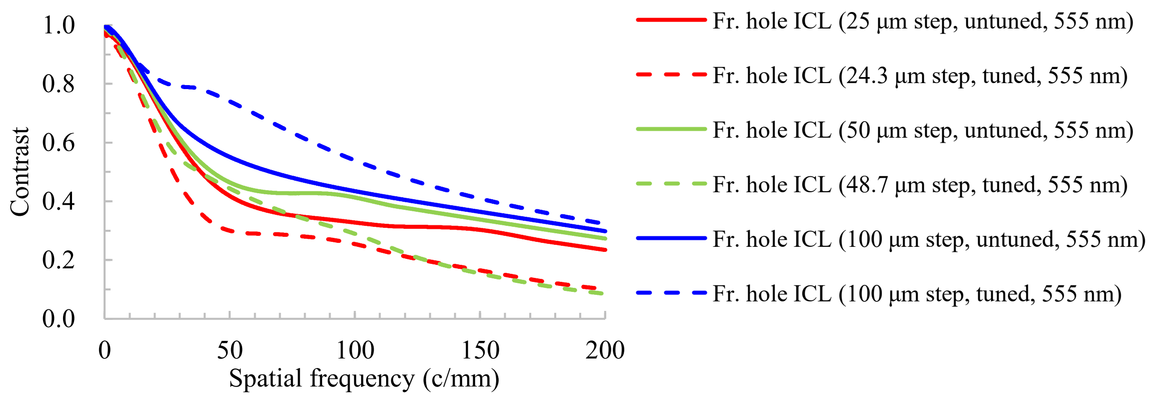

S1 Fig 2. FFT-based MTF comparison of Fresnel-inspired hole ICLs with different step heights under tuned and non-tuned conditions at a 4.5 mm artificial pupil (555 nm).

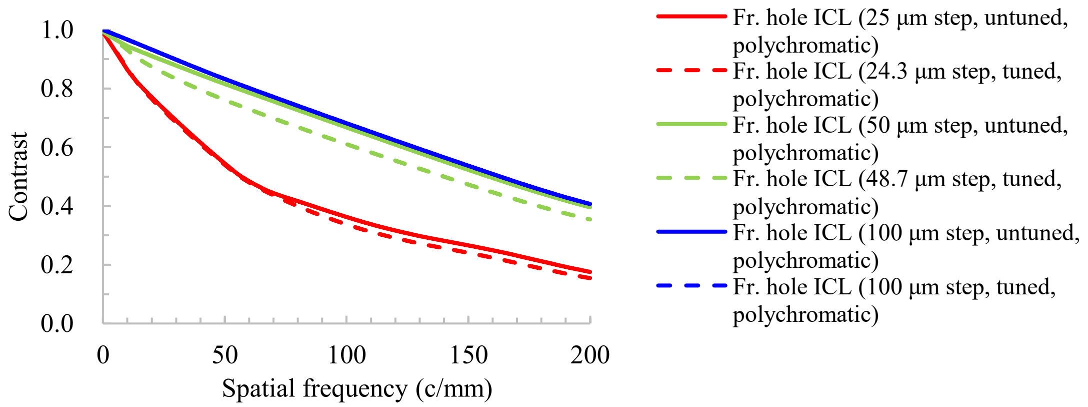

S1 Fig 3. FFT-based MTF comparison of Fresnel-inspired hole ICLs with different step heights under tuned and non-tuned conditions at a 3.0 mm artificial pupil (polychromatic).

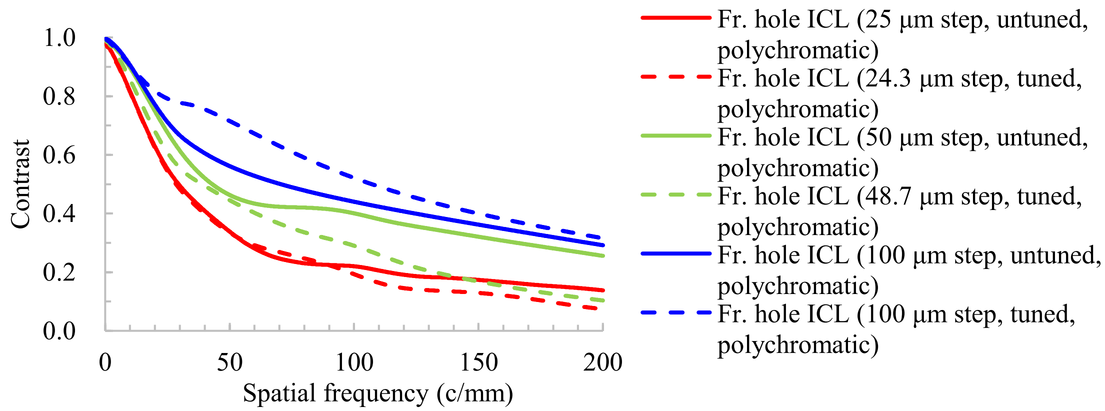

S1 Fig 4. FFT-based MTF comparison of Fresnel-inspired hole ICLs with different step heights under tuned and non-tuned conditions at a 4.5 mm artificial pupil (polychromatic).

S5. Summary of supporting analysis

Under the 3.0-mm pupil condition, satisfying the integer-wavelength (tuned) condition did not improve the MTF and, in some cases, resulted in a slight reduction. This behavior can be explained by the design concept of the hyperopic Fresnel ICL proposed in this study. The Fresnel structure employed here is not intended to optimize zone boundaries for interference control, as in diffractive lenses; rather, it is designed to reduce lens thickness by approximating a continuous refractive surface with a stepped geometry. Consequently, image quality is governed primarily by residual aberrations and pupil-diameter-dependent effects, rather than by coherent interference between adjacent zones.

In this study, optical design optimization was performed at a pupil diameter of 3.0 mm, and performance was subsequently evaluated at a larger pupil diameter of 4.5 mm without re-optimization. Consequently, under the 4.5 mm condition, the contribution of peripheral rays (light passing through the peripheral region) increases, and residual refractive errors—particularly defocus components—that were less pronounced at 3.0 mm become more prominent.

In the 100 µm step height design, applying the tuned condition altered the effective optical path distribution within the stepped structure, resulting in a slight redistribution of the pupil-averaged optical path difference (i.e., defocus component) in the peripheral pupil region. This redistribution may have partially canceled the residual defocus. Indeed, through-focus MTF analysis showed that the MTF peak position for the tuned condition shifted closer to the retinal plane, supporting this interpretation.

Furthermore, the 100 µm step height design has fewer steps, resulting in larger individual zones (ring-shaped regions). Therefore, under large pupil conditions such as 4.5 mm diameter, changes in optical path distribution occurring in the peripheral pupil region are more readily reflected and may manifest as shifts in the effective image plane position (defocus). In contrast, designs with smaller step heights (25–50 µm) have finer and more numerous steps, causing optical path length variations due to the steps to be more easily averaged across the pupil, making comparable defocus compensation effects less likely to occur.

In such designs, aligning the optical path difference between steps to an "integer number of wavelengths (integral multiples of 2π)" for a single design wavelength does not necessarily correspond to optimal image quality. Moreover, under polychromatic conditions closer to real viewing environments, the integer-wavelength condition cannot be simultaneously satisfied for all wavelengths, and wavelength-dependent phase errors are likely attenuated by pupil averaging. As a result, the contribution of inter-zone interference becomes relatively small, and under the simulation conditions of this study, the MTF and PSF evaluated by FFT showed little dependence on the tuned condition.

In summary, the results of this study support the interpretation that the Fresnel structure employed here functions more as a stepped approximation of a refractive surface rather than as a diffractive element exploiting interference, and that image quality is relatively robust to phase tuning of step heights.
